# The Effect of Erythropoietin Administration on the Serum Level of YKL-40, pro-BNP and IL-6 in Coronary Surgery Patients

**DOI:** 10.22037/ijpr.2020.112867.13993

**Published:** 2020

**Authors:** Mahnoosh Foroughi, Zohre Mohammadi, Masoud Majidi Tehrani, Mahmood Bakhtiari, Ali Dabbagh, Mostafa Haji Molahoseini

**Affiliations:** a *Cardiovascular Research Center, Shahid Beheshti University of Medical Sciences, Tehran, Iran. *; b *Clinical Research and Development Center, Shahid Modarres Hospital, Shahid Beheshti University of Medical Sciences, Tehran, Iran.*; c *Anesthesiology Research Center, Shahid Beheshti University of Medical Sciences, Tehran, Iran. *; d *Department of Immunology, School of Medicine, Shahid Beheshti University of Medical Sciences, Tehran, Iran.*

**Keywords:** Coronary artery bypass grafting, Erythropoietin, Inflammation, Ischemia, Reperfusion injury, YKL-40

## Abstract

Cardiopulmonary bypass and aortic clamping evokes the obligatory global myocardial ischemia and dysfunction with a significant inflammatory response. The discrepancy about cardioprotective effects of erythropoietin still exist. The aim of this study was to assess the clinical immunomodulatory effects of Erythropoietin (EPO) on serum inflammatory biomarkers (YKL-40, IL-6) and cardiac biomarkers, (pro-BNP, CK-MB and troponin). In this randomized double blind clinical trial, 132 patients admitted for elective coronary surgery with Cardiopulmonary Bypass (CPB) were randomly assigned in one of three groups: 1-group EPO-A (n = 35) infusion of 300 IU/Kg EPO after anesthesia induction and before undergoing CPB; 2- group EPO-CPB (n = 31) the same intervention during CPB; 3- placebo group (n = 66) saline infusion in the same volume. Cardiac enzymes and serum biomarkers were measured at intervals. There was a sharp increase in serum YKL-40 with a 24 h delay after CPB in all groups without significant difference. The increase in serum IL-6 was significant in EPO-CPB group compared with both other groups (*p *= 0.001 and *p* = 0.001, respectively). Serum pro-BNP reached maximum level 24 h after operation in all groups; in group A significantly less than others (*p *= 0.008). CK-MB increased significantly in all groups (*p* < 0.001), less prominently in CPB-A group (*p *= 0.03). EPO administration before induced ischemia may be cardioprotective in terms of cardiac biomarkers in patients undergoing CABG with CPB.

## Introduction

During cardiac surgical procedures, cardiopulmonary bypass (CPB) is one of the basic ingredients of the procedure. CPB is associated with a systemic inflammatory response and endothelial dysfunction due to artificial surfaces and surgical trauma, leading to ischemia-reperfusion (IR) injury (1-5). Aortic clamping and obligatory global cardiac ischemia affect the susceptible myocardial tissue to more damage. Though a number of measures have been used to defy inflammation, none has proved to be effective in an indisputable way yet (1, 4-8). 

Erythropoietin (EPO) has been acknowledged to release from kidney in response to hypoxic condition to stimulate erythropoiesis in bone marrow. Beyond the hematopoietic effects, a bulk of experimental studies suggests a cardioprotective role for EPO against IR injury. In limited clinical studies, this supportive role of EPO has been investigated during myocardial reperfusion states; both in surgical approach, i.e. coronary artery bypass graft (CABG) and in percutaneous coronary intervention (PCI); however, neither have yielded to a net definitive result (9, 10). EPO as a pleiotropic cytokine with anti-apoptotic and immune-modulatory effects has also been a matter of interest in clinical researches (11, 12).

YKL-40 or Chitinase-3-like protein 1 (CHI3L1), an inflammatory biomarker, has been recently quoted as an inflammatory biomarker in diagnosis, prognosis and cause of some diseases including cardiovascular pathologies. The name YKL-40 comes from the three main N-terminal amino acids, Y (tyrosine), K (lysine) and L (leucine), and also, molecular weight of 40 kDa (13, 14). Interleukin 6 (IL-6) as a pro-inflammatory biomarker and N-terminal pro-B type natriuretic peptide (pro-BNP) released from cardiomyocytes, could be used as a marker of ventricular dysfunction and cardiac surgery outcome (7, 15-18).

The aim of this randomized clinical trial was to assess the effects of EPO infusion in CABG patients by measuring serum levels of four biomarkers: YKL-40, IL-6, pro-BNP and CK-MB.

## Experimental

This study was approved by the local ethics committee and registered on the ClincalTrials.gov (Identifier: NCT02984111) and IRCT.ir (IRCTID: IRCT2016011621087N1). Written informed consent was obtained from all patients before participation. 

**Figure 1:**
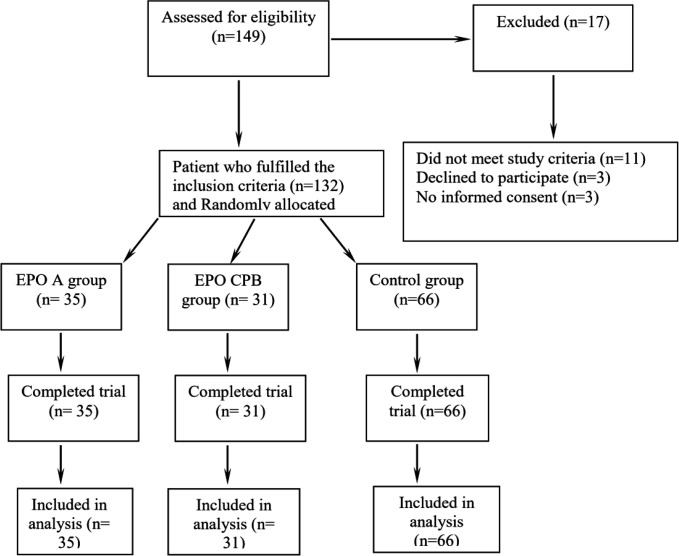
Study flow diagram. Among 149 patients who were included consecutively in this study, 17 patients were excluded and the remaining 132 patients were analyzed

In this prospective randomized double-blind placebo-controlled clinical trial, at first, 149 patients were included consecutively. Among them, 21 patients were excluded. The remaining 132 patients were randomly distributed into three study groups (Figure 1): 

EPO-A (i.e. EPO anesthesia): 300 IU/Kg EPO (PDpoetin®; Pooyesh Daru, Tehran, Iran) diluted in isotonic saline as a total volume of 50 ml and was systemically infused through central venous line in the time interval started after induction of anesthesia and finished just before initiating CPB; n* = 35*

EPO-CPB (i.e. EPO Cardiopulmonary Bypass): 300 IU/Kg EPO (PDpoetin®; Pooyesh Daru, Tehran, Iran) diluted in isotonic saline as a total volume of 50 mL and was systemically infused through central venous line in the time interval started just in the start of CPB and continued till aortic declamping; n = 31

P (Placebo): a total volume of 50 ml isotonic saline was infused in the time interval started after induction of anesthesia and continued till aortic declamping; n = 66

Sample size determination was based on similar previous trials investigating the protective effect of EPO in the adult coronary artery disease patients (19, 20). 

Inclusion criteria was defined as those patients in the study interval referring to the study center with two or more diseased coronary arteries scheduled for elective non-emergent CABG surgery with CPB and cardioplegic arrest for the first time; however, the patients presenting any of the following criteria were excluded: non isolated coronary surgery; combination of CAD and other cardiac pathologies; hemodynamic instability; recent MI; anemia (hemoglobin (Hb) < 11.5 g/dL); renal dysfunction (creatinine > 1.5 mg/dL); previous administration of EPO; hypercoagulable disorders; thromboembolic events; patient’s unwillingness to participate

All the patients, meeting the study criteria were randomly allocated in one of the three study groups; i.e. EPO-A; EPO-CPB and P, using a randomization list of computer program.

Coronary artery bypass using left internal mammary artery for LAD and saphenous vein grafts for other coronary arteries were done in the culprit lesions for all groups by one surgical team. All patients were treated according to a fixed protocol for general anesthesia, CPB set up, surgical techniques and myocardial preservation, that has been already described in detail (8, 21). Mean arterial pressure during CPB was maintained between 60-80 mmHg, adjustment was done with change in pump flow, dose of nitroglycerin or phenylephrine infusion and crystalloid solution. Hemoglobin concentration less than 7 mg/dL during CPB and less than 8 mg/dL before/after CPB were used as transfusion thresholds. All medical teams were blinded to group allocation; for this purpose, intravenous prepacked syringes were prepared while their contents were not disclosed to anyone but one of the colleagues. 

Clinical outcomes were the following items: duration of ventilator care; incidence of acute kidney injury (AKI) which was defined as elevation of serum creatinine more than 50% from baseline within 72 h after surgery; the amount of postoperative surgical bleeding; comparison of perioperative transfusion requirement; atrial fibrillation (AF) within 7 days after surgery; postoperative MI, stroke, hypertension crisis, and thromboembolic events

Laboratory endpoints were the trend of change in both systemic inflammatory and cardiac biomarkers by measuring their serum levels: YKL-40; IL-6; NT-pro-BNP; CK-MB; Troponin-I

Blood sampling for different biomarker measurements was accordingly based on the scheduled biomarker sampling time: before anesthesia induction (T1), at the end of surgery after protamine reversal (T2), the first (T3) and second (T4) postoperative day. The blood samples were centrifuged at 2500 rpm for 15 min within one hour after blood sampling, and the serum was stored at -20 °C until assayed. 

YKL-40 concentration was measured by a quantitative ELISA kit (Human Chitinase 3-liken1/YKL-40 ELISA Kit, Boster Immunoleader, Boster Biological Technology Co., Ltd. Pleasanton, CA, USA). The concentration of the IL-6 was measured by a colorimetric quantitative ELISA kit (Platinum ELISA, Affymetrix, eBioscince, USA). NT-pro-BNP measurement was done in serum using a commercially available two- site chemiluminescent immunometric assay (IMMULITE 2000 NT-pro-BNP, Siemens Healthcare, Mannheim, Germany), following the manufactures’ instructions.

Myocardial ischemia markers (CK-MB, troponin I) were measured before surgery as the baseline value (T0) and at the 6^th^ and 12^th^ postoperative hours after ICU arrival (T6 and T12, respectively). The CK-MB was measured by immunoinhibition assay (Anti CK-M. Immunoinhibition. Kinetic UV. Liquid, Bionik Diagnostic Systems, Tehran, Iran). On the other hand, troponin I was measured by chemiluminescence method (Ortho-clinical diagnostics a Johnson & Johnson company Vitros ECIQ immunodiagnostic, USA).

*Statistical Analysis*

Percentages were calculated for variables, whereas mean ± SD were expressed for the variables. One-way ANOVA followed by Tukey’s test was employed to investigate the mean difference between groups. Repeated measures of analysis of variance were also undertaken to investigate the mean differences of measured cardiac markers between the groups over time. All statistical analyses were performed using SPSS (version 11.5; SPSS Inc, Chicago, IL, USA). *p* values < 0.05 were considered statistically significant. 

## Results

*Baseline characteristic*

This study was performed in a tertiary University Hospital. The baseline characteristics and operative information are shown in Table 1. 

Preoperative medication and existing comorbidities were comparable between the three groups. The perioperative management was similar in the groups, with no differences in duration of surgery, administration of blood products, and inotrope drugs. The volume of bleeding had no statistical difference between all groups. There was one reoperation for postoperative bleeding in every group. The three study arms did not show any statistically significant difference in the serum creatinine level at the first 3 days postoperatively. None of the patients receiving EPO experienced the potential adverse effects (hypertension crisis, MI, stroke and thromboembolic events) until discharge.

*Laboratory outcome*

The mean concentration of the markers in the successive measurements are presented in Table 2. The baseline level of following markers had no difference between the three study groups. In comparison with the baseline values, all markers had statistically significant changes by CPB effect.

*Pro-BNP*

The repeated measure test showed the mean pro-BNP level had a significant difference between groups over time (*p *= 0.002).The increase in pro-BNP level began 24 h after CPB in all groups. Group EPO-A had a lower mean level in comparison with group EPO-CPB (*p* = 0.008). There was no significant difference between group EPO-A and control (*p* = 0.7), and also between groups EPO-CPB and control (*p* =0.058) at the first post operative day. One way ANOVA analysis revealed the mean level of pro-BNP in group EPO-A was significantly lower than the other groups in the first postoperative day (*p *< 0.001) (Table 2). 

*YKL-40*

After CPB, there was a mild increase in serum YKL-40. The peak level had a 24 h delay after operation and then decreased, but it did not return to basal laboratory range in 48 postoperative hours. Repeated measure test revealed serum YKL-40 had no significant difference between the groups over time (*p* = 0.375). Most of our patients had three vessel disease and the median serum YKL-40 in our study was 45(IQR: 23-68) ng/mL. There was a positive significant correlation between the number of diseased vessels and basal serum YKL-40 level (*p* = 0.037). Serum YKL-40 level in diabetic patients was higher than non-diabetic patients; however the difference was not significant (median (IQR):47.14 (17.55-59.03) ng/mL *vs*. 39.69 (23.63-72.81) ng/mL, *P* = 0.813).

*IL-6*

The serum IL-6 had significant difference between the groups after CPB (*p* =0.001 in T1 and P =0.042 in T2) (Table 2). *Post Hoc* test showed serum IL-6 in group EPO-CPB was significantly higher than in both group A and control in time T1 (*p* = 0.001 and *p* = 0.001, respectively). In time T2 there was a trend of increase in group EPO-CPB compared to control (*p* =0.044). Repeated measure test showed the serum IL-6 in group EPO-CPB was significantly higher than the other groups over time (*p* = 0.001 and *p* = 0.001, respectively).

*Cardiac enzymes*

All patients had an increase in postoperative CK-MB level. Repeated measure test showed the mean CK-MB level in group EPO-A was significantly lower than Placebo group (*P* = 0.03) and a trend for group EPO-CPB over time (*p* = 0.058). One-way ANOVA analysis showed a significant difference between study groups in T6 and T12 (*P* = 0.006 and *P* = 0.038, respectively). *Post Hoc* test showed mean CK-MB in group EPO-A was less than group EPO-CPB in T6 (*p* = 0.005) and less than Placebo group in T12 (*p* = 0.035). Although troponin I increased significantly in all groups, it had no significant difference between groups (*p* = 0.8). Table 2 depicts the postoperative level of cardiac enzymes and their changes.

*Secondary outcomes*

Postoperative AF was seen in all groups with no significant difference (*p *= 0.7). Among measured markers, there was a correlation between AF and pro-BNP at first postoperative day (*P* = 0.025). One-way ANOVA showed the ventilation time had a significant difference between groups (*p* = 0.008) (Table 3)*. Post Hoc* test showed the ventilation time in group EPO-A was significantly lower than both groups EPO-CPB and Placebo (*p* = 0.011 and *p* =0.041, respectively). There was no significant difference in term of the need for blood transfusion between the groups during operation and ICU and the incidence of AKI (One patient in each EPO group and 2 patients in placebo grpoup). All the patients recovered well from their surgery without experience of EPO adverse effects.

EPO-A: 300 IU/Kg EPO diluted in isotonic saline as a total volume of 50 mL and was systemically infused through central venous line in the time interval started after induction of anesthesia and continued till the start of CPB; n* = *35.

EPO-CPB: 300 IU/Kg EPO diluted in isotonic saline as a total volume of 50 mL and was systemically infused through central venous line in the time interval started just in the start of CPB and continued till aortic declamping; n* = *31

P: a total volume of 50 ml isotonic saline was infused in the time interval started after induction of anesthesia and continued till aortic declamping; n = 66

Values in study groups are presented as mean ± SD.

**Table 1:** The demographic and operative information of allocated patients to one of three arms of the study

**Variable, unit of measurement**	**Group EPO-A (n = 35)**	**Group EPO-CPB (n = 31)**	**Group P (n = 66)**	***P*** ** value**
Age, years	61.4 ± 10.1	60.4 ± 9.8	64 ± 9.5	0.34
Hypertension, n (%)	26 (74.3)	24 (77.4)	19 (61.3)	0.32
Diabetes mellitus, n (%)	13 (37.1)	11 (35.5)	12 (38.7)	0.96
Hyperlipidemia, n (%)	22 (62.9)	20 (64.5)	16 (51.6)	0.52
Previous myocardial infarction, n (%)	17 (48.6)	23 (74.2)	16 (51.6)	0.07
Current smoking, n (%)	19 (54.3)	16 (41.9)	13 (45.2)	0.58
Family history, n (%)	19 (48.6)	13 (74.2)	14 (51.6)	0.57
Erythrocyte sedimentation rate, mm/h	18.6 ± 12.7	19 ± 15.5	15.3± 13.7	0.53
Ejection fraction, %	51 ± 10	47 ± 11.1	49± 9.5	0.43
Aortic clamp time, minutes	48.4 ± 9.1	50.9 ± 1.6	47.3± 8.1	0.25
Pump time, minutes	93.6 ± 19.2	99.7 ± 15	88.9± 16.4	0.06
Bypass graft, n	3.25 ± 0.6	3.43 ± 0.7	3.16 ± 0.5	0.11

**Table 2:** The time courses of variation in the serum concentration of pro-BNP, CPK-MB and Troponin from baseline to postoperative period. Time points: T0 (before anesthesia induction), T1( after protamine reversal), T2(first postoperative day), T3(second postoperative day), T6 and T12 (6 and 12 h) after ICU arrival. Data are means ± SD. For description of groups, please see the undernote of Table 1

**Variable (unit of measurement)**	**Group EPO-A (n = 35)**	**Group EPO-CPB (n = 31)**	**Group P (n = 66)**	***P*** ** value**
**CPK-MB(U/L)**				
T_0_	15 ± 5	13 ± 4	18 ± 6	0.07
T_6_	33 ± 12	43 ± 17	42 ± 15	0.006
T_12_	37 ± 17	45 ± 15	53 ± 28	0.03
**Troponin (ng/mL)**				
T_0_	0.26 ± 0.32	0.20 ± 0	0.32 ± 0.55	0.49
T_6_	4.80 ± 3.76	5.58 ± 8.92	5.83 ± 6.41	0.98
T_12_	7.84 ± 7.22	6.56 ± 6.14	8.25 ± 7.36	0.63
**Pro-BNP (pg/mL)**				
T_0_	380 ± 718	551 ± 853	640 ± 887	0.31
T_1_	341 ± 623	556 ± 879	440 ± 622	0.26
T_2_	981 ± 1007	3030 ± 2991	1201 ± 630	0.000
T_3_	2209 ± 1717	3174 ± 3274	2362 ± 682	0.19
**YKL-40 (ng/mL)**				
T0	56 ± 48	43±39	60±43	0.34
T1	64 ± 50	70±60	62±45	0.86
T2	326 ± 330	40690	358±392	0.62
T3	252 ± 296	280±200	128±161	0.4
**IL-6 (pg/mL)**				
T0	1.5 ± 1.1	2.5 ± 1.3	2.4 ± 1.6	0.8
T_1_	64 ± 40	102 ± 49	49 ± 32	0.001
T_2_	23 ± 18	37 ± 42	19 ± 14	0.04
T_3_	8 ± 12	14 ± 22	8 ± 16	0.28

**Table 3:** Postoperative data in 3 study groups

**Variable (unit of measurement)**	**Group EPO-A (n = 35)**	**Group EPO-CPB (n = 31)**	**Group P (n = 66)**	***P *** **value**
Bleeding (ml)	440 ± 286	464 ± 194	513± 303	0.5
PRBC(unit)	0.5 ± 1.2	0.7 ± 0.8	0.6 ± 1.1	0.6
AF (%)	5(14%)	6(19%)	5(16%)	0.7
Time needed for Postoperative Mechanical Ventilation, hours	7.2 ± 2.8	11.3 ± 6.7	10.7 ± 5.9	0.008

PRBC, packed red blood cell; AF, atrial fibrillation. For description of groups, please see the undernote of Table 1.

## Discussion

This study demonstrated EPO infusion has a significant immunomodulatory effect on inflammatory biomarkers in patients undergoing cardiac surgery with CPB especially when administered just after start of anesthesia and before going on bypass. A less significant effect is seen if EPO is started during CPB.

Based on previous studies and our knowledge, this is the first clinical trial to evaluate the serum level of inflammatory glycoprotein YKL-40 as a biomarker of inflammation in cardiac patients undergoing CABG; this could be a hallmark of our study (13, 14, 22-27). 

YKL-40 (Chitinase-3-like Protein -1: a member of chitinase like proteins) is an inflammatory glycoprotein and a new biomarker of inflammation and cardiovascular disease (13, 22, 28). Besides, YKL-40 is an independent predictor or a prognostic biomarker of many cardiac events: coronary artery disease (CAD), number of diseased vessels, acute myocardial infarction (MI), angiographic lesion progression and cardiovascular mortality even in patients with stable CAD, fate of heart failure patients and overall mortality; also, statin treatment reduced serum YKL-40 level in CAD patients (13, 22, 25, 29-31). 

Previous studies have shown a 24 h delay between the beginning of the major stress such as MI and the rise of serum YKL-40 level to its peak and then decrescendo trend over time (13, 25, 28). This was similar to our study: a mild increase in serum YKL-40 at the end of surgery and 24 h later, it peaked more than 7 times the baseline and then decreased. Similarly, YKL-40 level increased up to 7-fold in acute MI setting and 4-fold in chronic stable CAD (29, 32). Possibly the inflammatory response due to CPB is at least as potent as MI to provoke YKL-40 release. There is currently no data in other kinds of surgery assessing the trend of YKL-40.

The patients who received EPO before CPB (group EPO-A) had less serum levels of IL-6, pro-BNP, and CK-MB with shorter mechanical ventilation time compared with those who received EPO during CPB or the control group. However, perioperative EPO administration was not associated with improvement in nearly all of the post-operative outcome measures of the study, including need for blood transfusion, chest tube drainage, AF, and AKI; except the time for postoperative mechanical ventilation.

Correlation between YKL-40 and IL-6 has been investigated before. YKL-40 expression could be upregulated in hypoxia (25, 32-34). IL-6 might be a reliable marker of activated inflammatory cascade and mortality. However, the serum IL-6 was significantly higher in EPO-CPB group than the other groups over time, which might be due to the poor correlation between YKL-40 values and IL-6. Keeping in mind that activation of inflammatory response is an inevitable part of CPB, this finding could possibly stress on the more sensitive role of YKL-40 in detection of inflammation compared to IL-6; this is a relatively novel finding in our study mandating more complementary research (14, 23, 25, 35). 

EPO has a number of anti-inflammatory effects as a pleiotropic cytokine, in addition to its well-known effect on erythropoiesis. Preclinical studies have indicated a broad variety of protective properties for EPO; however, when EPO has been used in clinical setting, controversial findings have been demonstrated regarding its potential anti-inflammatory and antioxidative effects (9, 10, 24, 26, 35-38). 

Moreover, EPO has been used during myocardial reperfusion states (either through CABG or percutaneous coronary intervention: PCI) (17, 19, 20, 23-25, 27, 30, 32, 35, 39). The experimental models emphasize the importance of intervention time, possibly due to narrow therapeutic EPO window. So, EPO administration before reperfusion injury (especially as short-term high dose EPO infusion) might be more effective with significant cardiac effects (9, 10, 19-21, 23, 24, 27, 29, 30, 36, 37, 39, 40). These studies support our findings in EPO-A group. 

Our findings on CK-MB and troponin I were not supported by some other previous studies. Mocini *et al*. showed the failure of preoperative EPO to protect against ischemic reperfusion injury using CK-MB and troponin I peaks in the first three postoperative days (27). However, the possible explanation for our different results may be due to the prolonged (daily) intervals between their measurements. Besides, time to peak and return to the normal level for CK-MB is significantly lower than Troponin I. On the other hand, in our study, the response of pro-BNP to CPB was similar in all groups; the latter finding is in concordance with a number of previous studies (6, 8, 18, 35, 36, 41). 

Our study could not demonstrate renal protective effects for EPO in cardiac surgical patients in contrast with some previous studies (19, 25). However, other results on this topic are somehow controversial which may be due to the enrollment of relatively few patients, different dosing and the timing of EPO administration in different studies (9, 10, 19, 32, 35, 36, 42, 43).

*Limitation*

several limitations of this study deserve mention. First, this was a single center study. We did not measure other inflammatory markers and had no long term follow up. The patient characteristics were similar in all groups, and our findings were rather homogeneous and uniform with relatively small power for external extrapolation. The serum level of the studied biomarkers was not corrected for hemodilution, though perioperative hemodilution was similar in all the patients. In addition, time points for collection of blood samples for analysis of inflammatory and cardiac markers were limited, and further measurement with closer intervals is suggested. 

## Conclusion

 myocardial damage during CPB is multifactorial with several complex interactions and pathways. To the best of our knowledge, the present study is the first clinical study that shows the effect of EPO on inflammatory biomarker levels (especially YKL-40) during cardiac surgery with CPB. The extent of the inflammatory response induced by CPB is at least as potent as MI to evoke YKL-40 level. Serum YKL-40 reaches peak level one day after CPB. However, further studies are suggested to assess the efficacy and safety of the EPO treatment and the prognostic value of YKL-40 in the patients undergoing CABG with CPB.

## Funding

the present study was supported by grants from the Research Department of the School of Medicine, Shahid Beheshti University of Medical Sciences, Tehran, Iran (no. 59658) and the Pooyesh Daru (no. 25957). The funders had no role in study design, data collection, statistical analysis, writing of the manuscript or decision to publish.
